# Comparative Efficacy and Tolerability of Three Treatments in Old People with Osteoporotic Vertebral Compression Fracture: A Network Meta-Analysis and Systematic Review

**DOI:** 10.1371/journal.pone.0123153

**Published:** 2015-04-13

**Authors:** Ling-Xiao Chen, Yu-Lin Li, Guang-Zhi Ning, Yan Li, Qiu-Li Wu, Jin-Xiu Guo, Hong-Yu Shi, Xiao-Bo Wang, Yong Zhou, Shi-Qing Feng

**Affiliations:** 1 Department of Orthopaedics, Tianjin Medical University General Hospital, 154 Anshan Road, Heping District, People’s Republic of China; University of Michigan, UNITED STATES

## Abstract

**Purpose:**

The question which kind of methods is most suitable for treating the old people for osteoporotic vertebral compression fracture is still discussed and pairwise meta-analyses cannot get hierarchies of these treatments. Our aim is to integrate the evidence to provide hierarchies of the comparative efficacy measured by the change of VAS (Visual Analogue Scale) and tolerability measured by incidence of new fractures and risk of all-cause discontinuation on three treatments (percutaneous vertebroplasty (PVP)、balloon kyphoplasty (BK) and conservative treatment(CT)).

**Methods:**

We performed a Bayesian-framework network meta-analysis of randomized controlled trials (RCTs) to compare three treatments for the old people with osteoporotic vertebral compression fracture. The eligible RCTs were identified by searching Amed, British Nursing Index, Embase, Pubmed, the Cochrane Central Register of Controlled Trials (CENTRAL), Google scholar, SIGLE, the National Technical Information Service, the National Research Register (UK) and the Current Controlled Trials databases. Data from three outcomes (e.g. VAS, risk of all-cause discontinuation and incidence of new fractures) were independently extracted by two authors.

**Results:**

A total of five RCTs were finally included into this article. PVP and BK significantly decreased VAS when compared with CT. BK had a significantly lower risk of all-cause discontinuation contrast to CT. Three treatments (BK, PVP and CT) had no significant differences in the incidence of new fractures.

**Conclusions:**

PVP may be the best way to relieve pain, CT might lead to the lowest incidence of new fractures and BK might had the lowest risk of all-cause discontinuation in old people with osteoporotic vertebral compression fracture. More large-scale and longer duration of follow-up studies are needed.

## Introduction

Vertebral compression fracture is a common complication in people with osteoporosis, especially in old people. In Europe, the occurence rate of morphometric fracture was 10.7/1,000 person year (pys) in female and 5.7/1,000 pyr in male respectively, which indicated the occurrence rate increased with the age [1]. Balloon kyphoplasty (BK), vertebroplasty (PVP) and conservative treatment (CT) are three main treatments for this disease. PVP was first described in 1980 to treat vertebral hemangioma [2]. Until now, it is widely used in patients with back pain and vertebral compression fracture [3, 4]. BK was a relatively new technology which reduced pain by using inflatable bone tamp to compact the cancellous bone [5, 6]. The question which treatment should be preferred was debated. Previous pairwise meta-analyses could not get hierarchies of these treatments because some treatments had not been compared one by one [7–10]. In addition, the number of included RCTs was limited, which led to some potential interferences on conclusions.

We aimed to compare the efficacy and tolerability of three treatments (BK, PVP and CT) for osteoporotic vertebral compression fracture in old people. Our intention was to provide hierarchies of the comparative VAS (Visual Analogue Scale), risk of all-cause discontinuation and incidence of new fractures on three treatments.

## Methods

### Criteria for considering studies

We only included RCTs, which compared VAS, risk of all-cause discontinuation and incidence of new fractures of three main interventions (BK, PVP and CT) in old people with osteoporotic vertebral compression fracture.

Studies were included in the systematic review if they met the criteria: (1) Osteoporotic vertebral compression fracture; (2) RCTs; (3) Ages > 60; (4) BK, PVP, CT, optimum pain treatment and optimal medical therapy.

Trials were excluded if they: (1) were abstracts, letters, or meeting proceedings; (2) had repeated data or did not report outcomes of interest; (3) had a duration of follow-up < 6 months; (4) were retrospective design.

### Search methods and study selection

We searched Amed (From 1985 to July 2014), British Nursing Index (From 1985 to July 2014), Embase (From 1974 to July 2014), Pubmed (From 1966 to July 2014), the Cochrane Central Register of Controlled Trials (CENTRAL) (The Cochrane Library, most recent issue), Google scholar, SIGLE (System for Information on Grey Literature in Europe), the National Technical Information Service, the National Research Register (UK) and the Current Controlled Trials databases. Keywords and MeSH terms including “balloon kyphoplasty”, “vertebroplasty”, “conservative treatment”, “optimum pain treatment”, “optimal medical therapy”, “osteoporotic**”** and “vertebral compression fracture” were used in the search strategy. We also viewed each reference list for any ignored papers.

Two review authors independently made the selection based on title and abstract. Any disagreement between review authors was resolved by discussion. If there were still some debates, a further reviewer and expert (Feng) was consulted.

### Data collection and quality assessment

One review author screened the paper and removed ineligible references, moreover, contacted corresponding authors if some other information was needed. Information including trial name, sample size, comparators, country, study design, maximum follow-up, effect sizes and p value for three outcomes were extracted for each included study. We would contact the original author to ask for any missing information. If the article could not provide standard deviations (SDs) and we could not get data from the author, there were two ways to solve this problem. The one was to calculate of the missing SDs if some other data were supplied, such as, MDs, p value and number of patients. The other was to manually measure by graphs presented in article.

We used the Cochrane risk of bias tool to assess risk bias of included studies [11]. The tool has seven domains including random sequence generation, allocation concealment, blinding of participants and personnel, blinding of outcome assessment, incomplete outcome data, selective reporting and other bias. The classification of the judgment for each domain was low risk of bias, high risk of bias, or unclear risk of bias and two authors independently evaluated the risk of studies.

### Data synthesis and analysis

Data were extracted and entered into Excel by two reviewers. Then, they checked the data of each other. When there were anything different, they returned to the original article to find the right answer. For continuous data (e.g.VAS), the standardized mean differences (SMDs) with 95% confidence interval (CI) for direct comparisons or 95% credible intervals (CrI) for indirect comparisons was used. Dichotomous data were used for reporting on: risk of all-cause discontinuation and incidence of new fractures. All-cause discontinuation was a relatively objective outcome to measure an intervention’s efficacy and tolerability which had real-world applicability because it could be easily got in the follow-up. Physicians or patients might discontinue the follow-up when they thought the intervention was ineffective or intolerable in long duration of follow-up [12]. Ballon kyphoplasty is a relatively new technology which lacks evidence of long duration of follow-up, however, all-cause discontinuation can supply some information about efficacy and tolerability in the long duration of follow-up. It was reported as odds ratios (ORs) with 95%CI for direct comparisons or 95%CrI for indirect comparisons.

Considering clinical diversity, methodological diversity and statistical heterogeneity, there was no obvious difference among five included studies (S1 File) which meant the three treatment effects originating from one common distribution.

Our network was a closed triangular circular network including both direct and indirect evidences. The model (which was proposed by Anna Chaimani, downloaded from www.mtm.uoi.gr) we used was fit for all kinds of networks [13, 14]. To the only one triangular circular, we used ifplot command proposed by Anna Chaimani to evaluate the consistency of direct and indirect estimates [14]. The results indicated direct and indirect estimates were consistent (S1 Fig).

Firstly, we made pairwise meta-analyses for studies which directly compared different treatments by using Stata software (version 12.0, StataCorp, College Station, TX). DerSimonian and Laird random effects model was used. The pooled estimates of ORs or SMDs and 95% CI of three outcomes (risk of all-cause discontinuation, VAS and incidence of new fractures) were shown. Chi-square test and I-square test were used for testing heterogeneity among the studies.

Then network meta-analysis was performed by using WinBUGS (version 1.4.3, MRC Biostatistics Unit, Cambridge, UK) with random effects models developed by Chaimani (downloaded from www.mtm.uoi.gr). We used the Markov Chains Monte Carlo (MCMC) method to get results, which were reported as posterior distribution median with 95% CrI. Non-informative uniform and normal prior distributions were performed to fit the model [15]. For each analysis, we used three MCMC chains, each of which includes 100,000 iterations with initial 50,000 discarded as the burn-in period in advance [16]. To rank the treatments, we used two ways. Firstly, we used posterior probabilities of outcomes to calculate probabilities of treatment ranking. Secondly, we used the surface under the cumulative ranking probabilities (SUCRA) to indicate which treatment was the best one.

The funnel plot was used to identify possible publication bias if the number of studies was larger than 10. The sensitivity analysis was performed by excluding researches with different duration of follow-up and studies with high risk of bias. There was no protocol about this research.

## Results

### Study identification and selection

**Fig 1:**
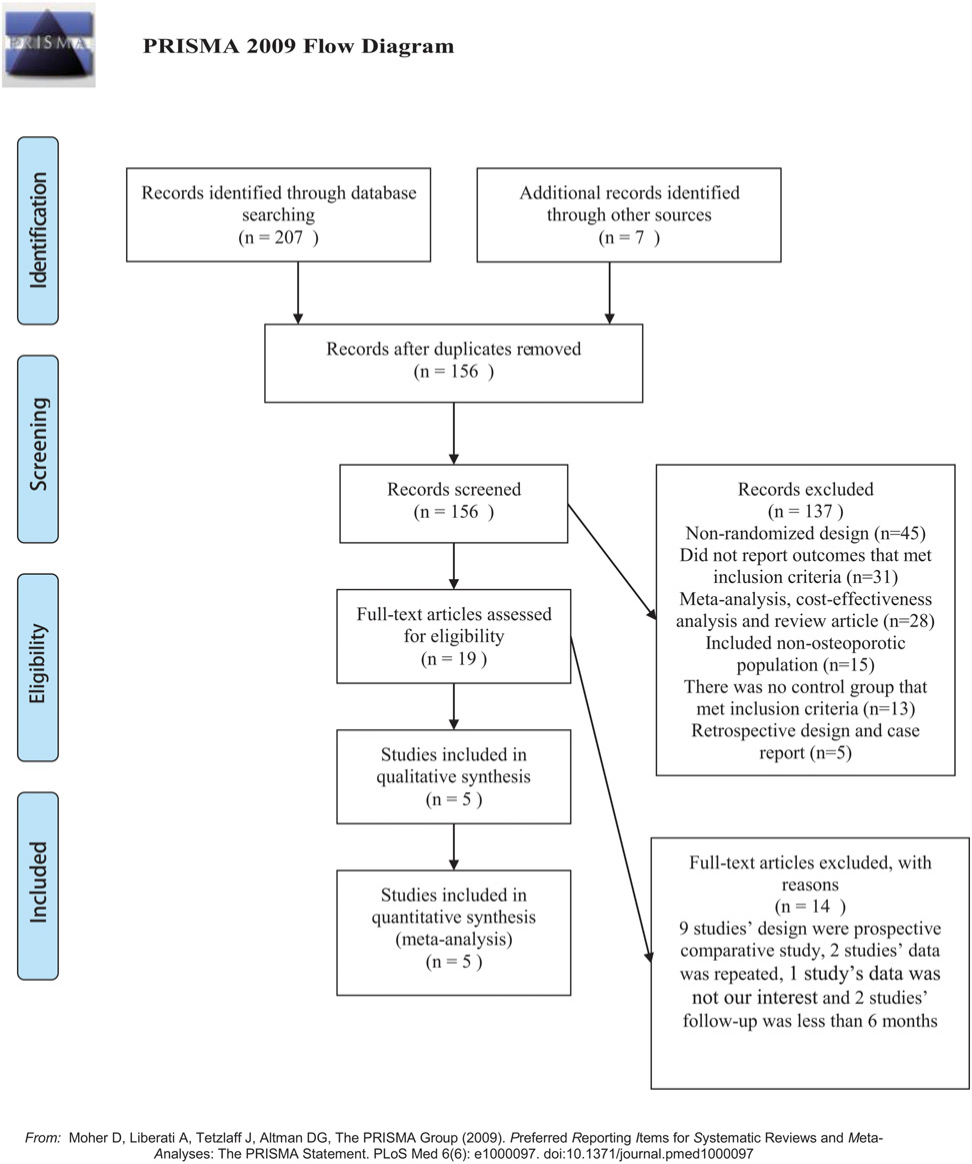
Flow diagram. For more information, visit www.prismastatement.org.

The PRISMA flow diagram of studies selection was depicted in Fig 1. The search was performed on July 12^th^, 2014 and identified 207 references in the primary search and 7 through other sources. After removal of 78 duplicate references, 156 records were screened. 19 publications were eligible for inclusion, and others were not selected for various reasons (e.g., studies without a control group and publications not related to osteoporotic vertebral compression fracture) (S2 File). In total, 19 studies were included in the narrative review and data from 5 of these studies [17–21] were included in the meta-analysis. 14 studies were excluded, because 9 studies’ design[22–30] were prospective comparative study, 2 studies’ data [31, 32]was repeated, 2 studies’ follow-up was less than 6 months [33, 34] and 1 study’s data [35] was not related.

### Study characteristics

Table 1 provided a summary of the studies in the review. A total of 777 participants were included in the article. Study sample size ranged from 49 to 300. All studies were RCTs directly comparing one treatment to another. These studies were published between 2009 and 2012 year. Five studies reported VAS as an outcome: three of them were on PVP vs CT, the results of which showed PVP significantly decreased VAS comparing with CT; one was on BK vs CT, the result of which indicated BK significantly decreased VAS; the last one was on PVP vs BK and there was no significant difference between two interventions. Four studies used risk of all-cause discontinuation as an outcome: three was PVP vs CT and no significant difference was found; one was BK vs CT and BK had significantly lower risk of all-cause discontinuation. Incidence of new fractures was the third outcome used by three studies: two was PVP vs CT, one was BK vs CT and there was no significant difference among three interventions.

**Table 1:** Study Characteristics of included studies.

Trial	Sample size	Comparators	Country	Maximum follow-up	Study design	VAS(SMD with 95%CI)	INF(OR with 95%CI)	AAD(OR with 95%CI)
Wardlaw,2009	300	BK vs NSC	Eight^a^	12 months	RCT	-1.13(-1.38,-0.89); P<0.0001	1.46(0.8–2.7);P = 0.22	0.44(0.21–0.95);P = 0.036
Klazen,2010	202	PVP vs CT	Two^b^	12 months	RCT	-1.48(-1.79,-1.17); P<0.0001	1.27(0.25,6.38);P = 0.769	0.65(0.35–1.2);P = 0.165
Rousing,2010	49	PVP vs CT	Denmark	12 months	RCT	-0.64(-1.21,-0.07); P = 0.028	2.4(0.95,6.06);P = 0.065	0.7(0.11,4.58);P = 0.707
Liu,2010	100	PVP vs BK	Taiwan	6 months	RCT	0.15(-0.25,0.54); P = 0.469	/	/
Blasco,2012	125	PVP vs CT	Spain	12 months	RCT	-3.31(-3.85,-2.76); P<0.0001	/	1.34(0.58,3.05);P = 0.493

PVP: Percutaneous vertebroplasty; BK: Balloon kyphoplasty; NSC: Non-surgical care; CT: Conservative treatment

Non-surgical care was also thought to be Conservative treatment.

VAS: Visual Analogue Scale; INF: Incidence of new fractures; AAD: All-cause discontinuation

SMD: standardized mean difference; OR: odds ratio

^a^: UK, Netherland, Belgium, Germany, Australia, France, Sweden and Italy

^b^: Netherland and Belgium

### Risk of bias in included studies

**Fig 2:**
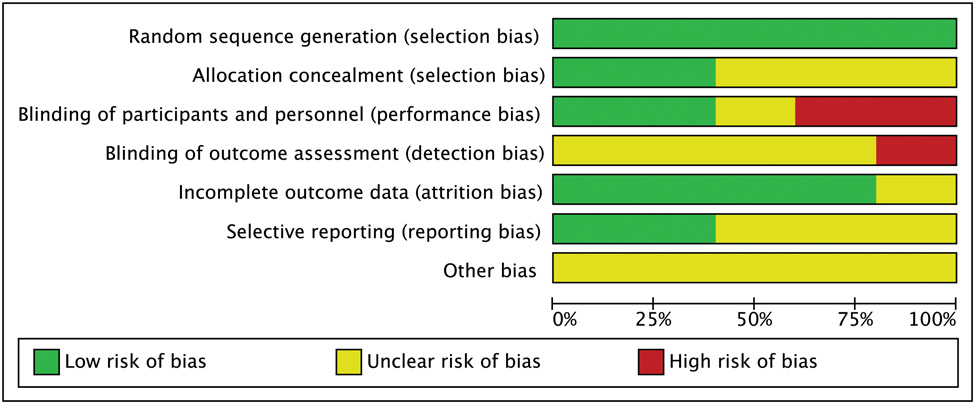
Risk of bias graph: review authors’ judgements about each risk of bias item presented as percentages across all included studies.

**Fig 3:**
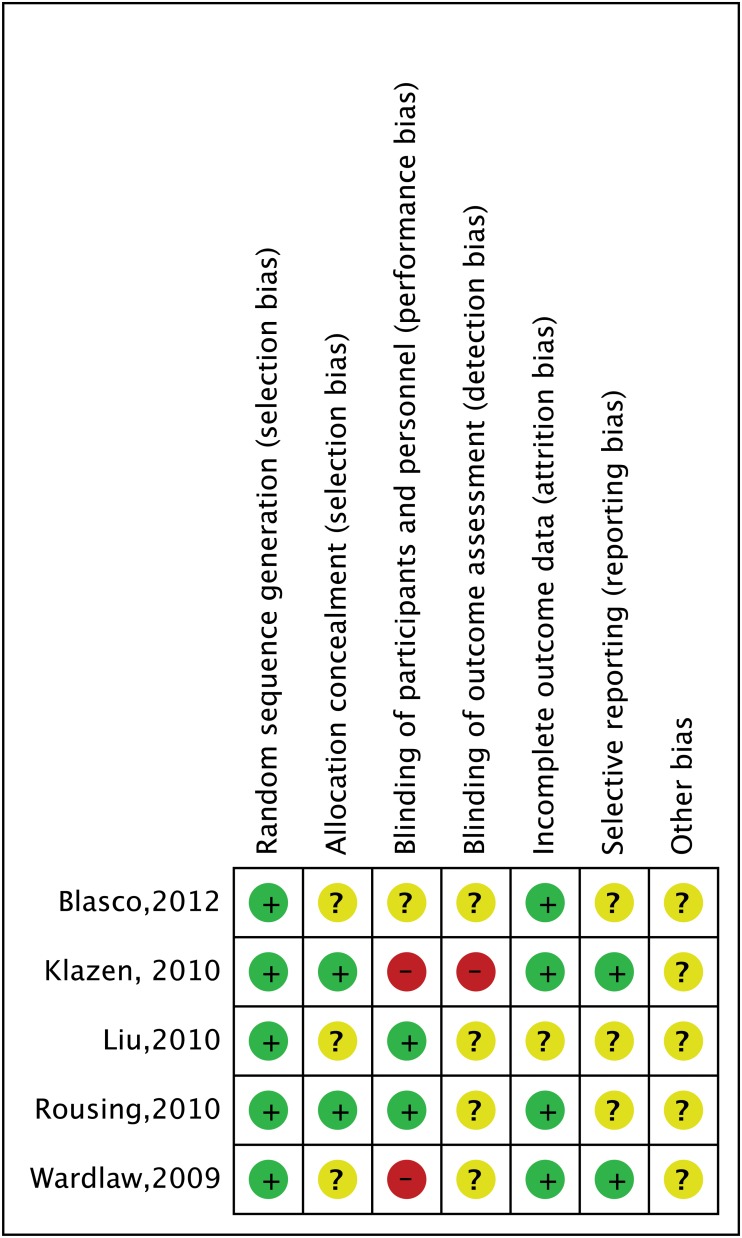
Risk of bias summary: review authors’ judgements about each risk of bias item for each included study.

Fig 2 and Fig 3 showed risk bias in all 5 studies. Five studies (100%) described random sequence generation [17–21]. Two studies (40%) described adequate allocation concealment [18, 20]. Two studies (40%) described blinding of participants and personnel [19, 20]. Two studies had high risk of bias about blinding of participants and personnel because one was an open-label trial and the other was explicitly described no blinding of participants and personnel in method section [18, 21]. Considering one research was an open-label trial, we thought it had a high risk of bias about blinding of outcome assessment [18]. Four studies (80%) had a low risk of incomplete outcome data [17, 18, 20, 21]. Although some researches had dropout, the effect of intervention was not affected due to due to the small scale of dropout. Three studies (60%) had low risk of selectively reporting results [18, 20, 21].

### Effects of treatments on the change of VAS

A total of 241 patients (31.1%) were assigned to PVP therapy, 199 (25.6%) to BK therapy and 337 (43.4%) to CT therapy.

**Fig 4:**
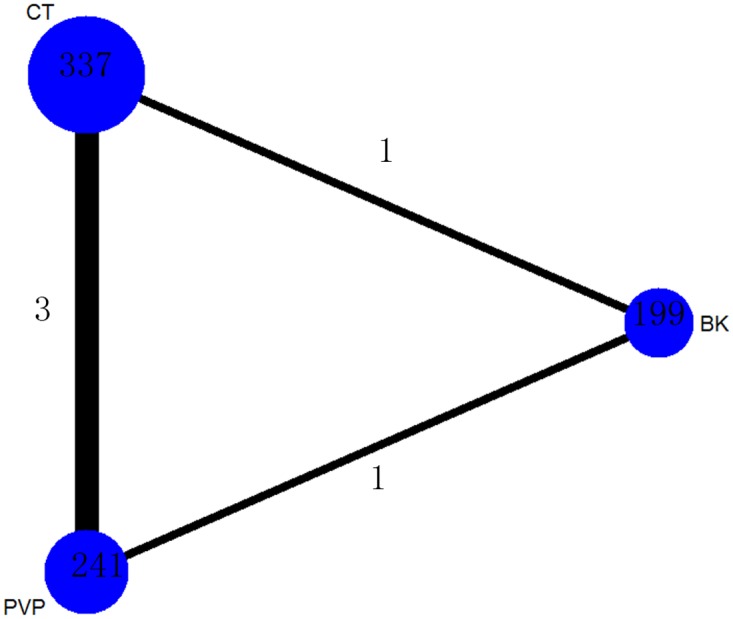
Network of treatment comparisons for VAS. The network plot shows direct and indirect comparisons. The size of the nodes represents the total sample size of treatments. The lines’ thickness corresponds to the number of trials that compare each other. PVP: Percutaneous vertebroplasty; BK: Balloon kyphoplasty; CT: Conservative treatment.

**Fig 5:**
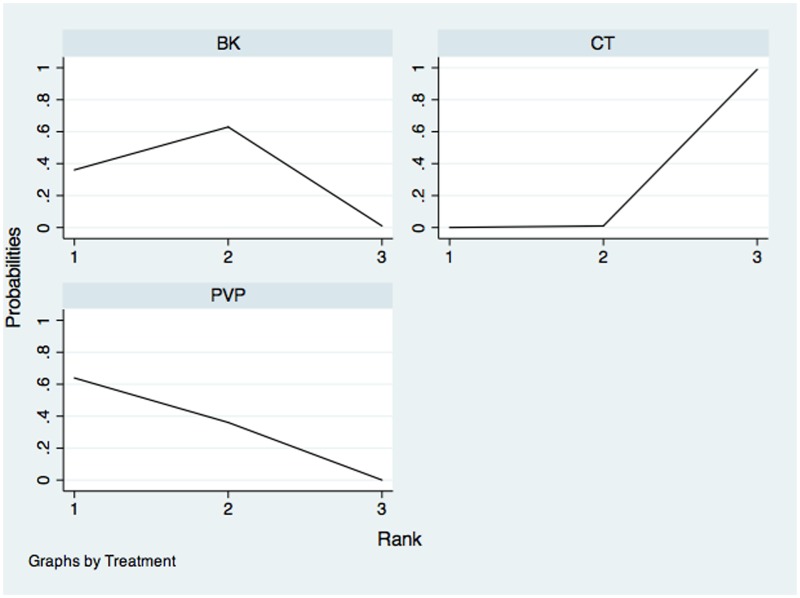
Ranking of treatment strategies based on probability of their effects on outcome of VAS. PVP: Percutaneous vertebroplasty; BK: Balloon kyphoplasty; CT: Conservative treatment.

The network of comparisons on VAS was shown in Fig 4. Table 2 provided hierarchies of effect size on VAS. We also made ranking graph of distribution of probabilities on VAS in Fig 5. The direct and indirect comparisons indicated PVP and BK significantly decreased VAS compared with CT. Based on SUCRA, PVP (0.8195) ranked the first, the second was BK (0.6753) and the last was CT (0.005222).

**Table 2:** VAS of different treatments.

PVP	0.15(-0.25–0.54)	**-1.81(-3.1–0.47)**
-0.22(-1.3–0.8)	BK	**-1.1(-1.4–0.89)**
**-1.71(-2.4–0.95)**	**-1.5(-2.6–0.4)**	CT

For VAS, standard mean differences (SMDs) lower than 0 favour the column-defining treatment. Direct comparsions were shown in the upper right. Indirect comparsions were shown in the bottom left. The number which was painted by a style of overstriking indicated there was a significant difference between two treatments. PVP: Percutaneous vertebroplasty; BK: Balloon kyphoplasty; CT: Conservative treatment.

### Effects of treatments on the risk of all-cause discontinuation

A total of 191 patients (28.2%) were assigned to PVP therapy, 149 (22%) to BK therapy and 337 (43.4%) to CT therapy.

**Fig 6:**
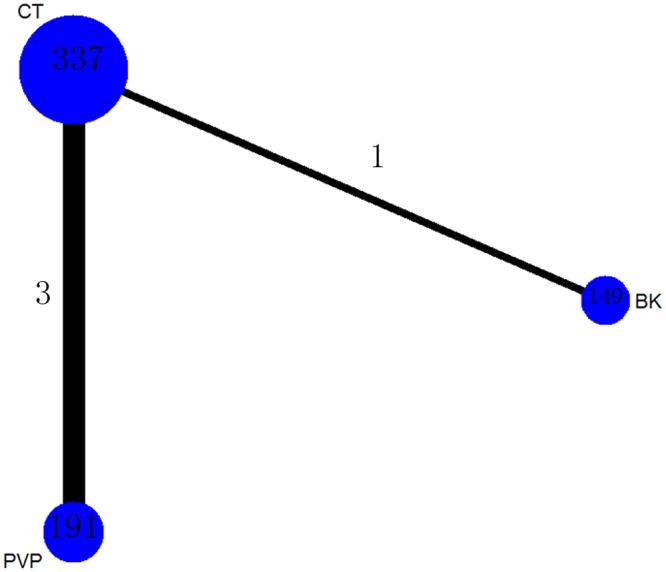
Network of treatment comparisons for risk of all-cause discontinuation. The network plot shows direct and indirect comparisons. The size of the nodes represents the total sample size of treatments. The lines’ thickness corresponds to the number of trials that compare each other. PVP: Percutaneous vertebroplasty; BK: Balloon kyphoplasty; CT: Conservative treatment.

**Fig 7:**
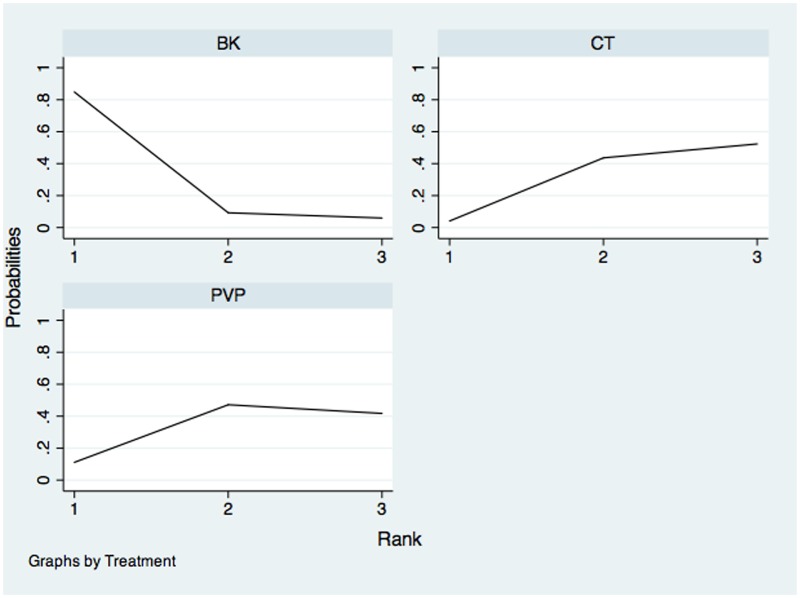
Ranking of treatment strategies based on probability of their effects on outcome of all-cause discontinuation. PVP: Percutaneous vertebroplasty; BK: Balloon kyphoplasty; CT: Conservative treatment.

The network of comparisons on the risk of all-cause discontinuation was shown in Fig 6. Table 3 provided hierarchies of effect size on all-cause discontinuation. We also made ranking graph of distribution of probabilities on all-cause discontinuation in Fig 7. Only direct comparison showed BK had a significantly lower risk of all-cause discontinuation compared with CT. On the basis of SUCRA, BK (0.8943) ranked the first, the second was PVP (0.3469) and the last was CT (0.2588).

**Table 3:** All-cause discontinuation of different treatments.

PVP	/	0.9(0.53–1.5)
3(0.67–7.8)	BK	**0.44(0.21–0.95)**
1.04(0.5–1.9)	0.53(0.15–1.2)	CT

For all-cause discontinuation, odds ratios (ORs) higher than 1 favor the column-defining treatment. Direct comparsions were shown in the upper right. Indirect comparsions were shown in the bottom left. The number which was painted by a style of overstriking indicated there was a significant difference between two treatments. PVP: Percutaneous vertebroplasty; BK: Balloon kyphoplasty; CT: Conservative treatment.

### Effects of treatments on the incidence of new fractures

A total of 90 patients (18.9%) were assigned to PVP therapy, 149 (31.4%) to BK therapy and 236 (49.7%) to CT therapy.

**Fig 8:**
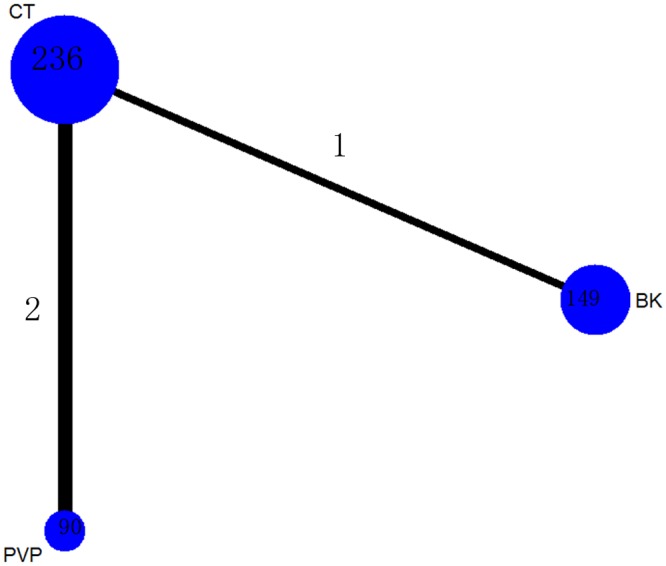
Network of treatment comparisons for incidence of new fractures. The network plot shows direct and indirect comparisons. The size of the nodes represents the total sample size of treatments. The lines’ thickness corresponds to the number of trials that compare each other. PVP: Percutaneous vertebroplasty; BK: Balloon kyphoplasty; CT: Conservative treatment.

**Fig 9:**
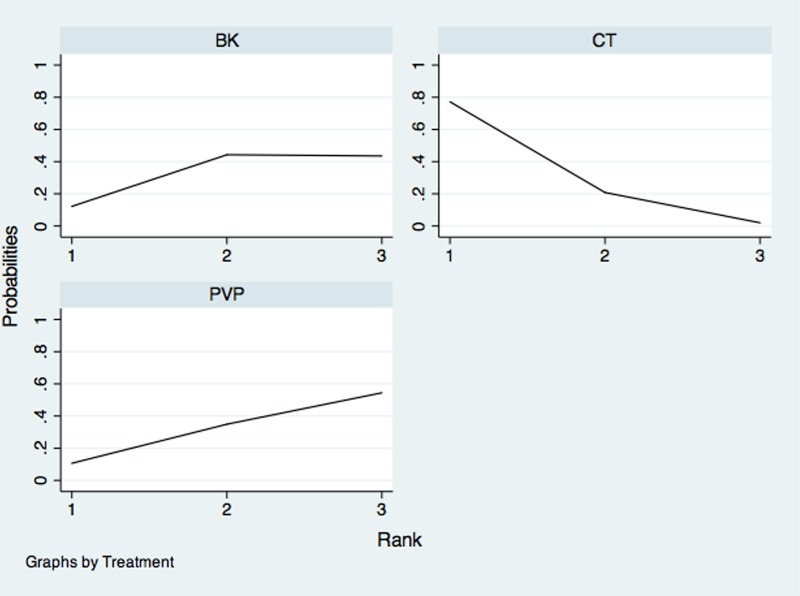
Ranking of treatment strategies based on probability of their effects on outcome of new fractures. PVP: Percutaneous vertebroplasty; BK: Balloon kyphoplasty; CT: Conservative treatment.

The network of comparisons on the incidence of new fractures was shown in Fig 8. Table 4 provided hierarchies of effect size on the incidence of new fractures. The ranking graph of distribution of probabilities on the incidence of new fractures was shown in Fig 9. Both direct and indirect comparisons showed three treatments had no significant differences on the incidence of new fractures. On the basis of SUCRA, CT had the lowest probability to have new fractures (0.8759), the second was BK (0.3432) and the third was PVP (0.2809).

**Table 4:** New fractures of different treatments.

PVP	/	2.05(0.92–4.6)
1.6(0.28–4.3)	BK	1.46(0.8–2.7)
2.4(0.73–5)	2.2(0.7–4.7)	CT

For new fractures, odds ratios (ORs) higher than 1 favor the column-defining treatment. Direct comparsions were shown in the upper right. Indirect comparsions were shown in the bottom left. The number which was painted by a style of overstriking indicated there was a significant difference between two treatments. PVP: Percutaneous vertebroplasty; BK: Balloon kyphoplasty; CT: Conservative treatment.

### Other outcomes

#### Wardlaw2009

The main outcome measures were short-form (SF)-36 physical component summary (PCS) scale, EQ-5D, RDQ, restricted activity days and bed rest. The improvement in SF-36 PCS score favored BK group (score from baseline to 1 year: 3.5, 1.6–5.4, P = 0.0004). BK group significantly increased EQ-5D score compared with non-surgery group (score from baseline to 1 month, P = 0.0003; score from baseline to 12 month, P = 0.0252). The RDQ score preferred BK group rather than non-surgery group (score from baseline to 1 month, P<0.0001; score from baseline to 12 month, P = 0.0012). BK group significantly decreased restricted activity days and bed rest about 2.9 days per 2 weeks at 1 month (P = 0.0004), however, there was no significant difference between two groups in 12 months (P = 0.0678).

#### Klazen2010

The main outcome measures were cost-effectiveness, EQ-5D, RDQ and QUALEFFO. PVP group significantly increased EQ-5D, RDQ and QUALEFFO scores compared with CT group (P<0.05, P<0.0001, P<0.0001; respectively). For cost-effectiveness, if the society spent €30 000 or more per Quality Adjusted Life Years got, PVP might be a better choice.

#### Rousing2010

The main outcome measures were SF-36, Dallas Pain Questionnaire (DPQ), EQ-5D, Barthel and a modified mini-mental state examination (MMSE). CT showed a significantly better outcome in DPQ after 3 months and PVP group had a significant better outcome in the Barthel index after 12 months. PVP group significantly increased EQ-5D score at 3 months (P = 0.04) under the unbalanced baseline of patients (P = 0.05). There were no significant differences in SF-36, MMSE, EQ-5D and RDQ at every time points except the time mentioned.

#### Liu2010

Vertebral body height and kyphotic wedge angle were the indication of the main outcome. BK group significantly increased vertebral body height and decreased kyphotic wedge angle compared with PVP group (P<0.0001).

#### Blasco2012

The main outcome measure was the QUALEFFO. PVP group significantly increased QUALEFFO scores compared with CT at every time points except at and after the 6 months. The further analysis showed the differences between two groups were attributed to the physical activity domain of QUALEFFO.

### Reporting biases and sensitivity analyses

Publication bias was not assessed because the number of studies was limited (<10). Sensitivity analyses where one study with a different duration of follow-up (S1 Table) and a study with high risk of bias (S3 File) were excluded did not change the result.

## Discussion

### Summary of main results

The network meta-analysis provided hierarchies for the VAS, risk of all-cause discontinuation and incidence of new fractures in old patients with osteoporotic vertebral compression fracture treated with different methods, which had advantages in the comparison with traditional meta-analyses [36–38]. The meta-analysis indicated that: (1) PVP and BK significantly decreased VAS compared with CT; (2) Only direct comparison showed BK had significantly lower risk of all-cause discontinuation contrast to CT; (3) Three treatments had no significant differences on the incidence of new fractures; (4) For decreasing VAS, the rank on treatments was: PVP, BK and CT; (5) For reducing incidence of new fractures, the rank on treatments was: CT, BK and PVP; (6) For lowering risk of all-cause discontinuation, the rank on treatments was: BK, PVP and CT.

### Strengths and weaknesses

There were some strengths in this article: (1) we used comprehensive search strategy to minimize possibilities of publication bias; (2) the article referred to the results of direct and indirect comparisons; (3) the posterior probabilities of outcomes and SUCRA were used to distinguish the subtle differences among three treatments; (4) only RCTs that described random sequence generation were included in this article.

However, the results of the review should be interpreted under some limitations. First, both the number of included studies and the sample size was so small that realistic assessment of the outcomes could not be made. Second, some study characteristics such as sex, performance bias and detection bias might be potential obstacles to the outcomes of our article. In addition, due to the inconformity about duration of follow-up, there was substantial heterogeneity. So we made a sensitivity analysis by excluding one study with a different duration of follow-up. The results showed there was no difference. Therefore, we thought the duration of follow-up might not influence the results. What’s more, the Klazen study and the Wardlaw study were not blinding to participants and personnel. Moreover, Blasco study, Liu study and Wardlaw study did not describe allocation concealment so performance and detection biases might occur, which made the outcome unreliable and we should cautiously explain the result. Finally, due to the difference in indications in included studies, the results might be influenced and we had better carefully deal with the results.

### Agreements and disagreements in the current literature

A previous meta-analysis indicated BK significantly decreased pain comparing with PVP for the long-term VAS scores (MD -1.06, 95%CI -2.01 to -0.1, p = 0.03, I^2^ = 98%)[9]. However, Dan Xing and colleagues showed there was no significant difference between BK and PVP for the long-term VAS scores (MD -0.99, 95%CI -2.29 to 0.31, p = 0.14, I^2^ = 98%)[10]. Jintao Liu et al. found PVP significantly decreased pain in addition to CT for VAS scores after more than 6 months (MD -1.59, 95%CI -2.14 to -1.04, p<0.01, I^2^ = 46%)[8]. The subgroup analyses demonstrating the significant difference between PVP and CT was mainly attributed to the pooled analysis between PVP and non-operative treatment (MD -1.76, 95%CI -2.34 to -1.18, p<0.01, I^2^ = 0%), on the other hand, the result of comparison between PVP and sham injection showed no significant difference (MD 0.00, 95%CI -1.77 to 1.77). Stevenson et al. told us basically both BK and PVP decreased pain compared with control group [39]. What’s more, there was no significant difference in the incidence of new fractures. Our network analysis showed there was no significant difference in the change of VAS between BK and PVP, meanwhile, PVP and BK significantly decreased VAS compared with CT. The outcomes agreed with the study by Dan Xing, Stevenson and Jintao Liu. For incidence of new fractures, similar to reports from previous meta-analysis [7, 9, 10, 39]. Our article provided new comparison (e.g. BK vs CT) on VAS and outcome of risk of all-cause discontinuation that other reviews had not mention.

### Conclusions

The data based on the relatively small numbers suggest that PVP may be the best way to relieve pain, CT might lead to the lowest incidence of new fractures and BK might had the lowest risk of all-cause discontinuation in old people with osteoporotic vertebral compression fracture. However, because of limited sample size and study numbers, more large-scale and longer duration of follow-up studies are needed to examine the effect of three main interventions (BK, PVP and CT).

Currently, four studies is ongoing about three treatments, three of which are about PVP and CT, and the last one is about BK and CT [40–43]. The results of ongoing researches might provide evidence with higher quality than before.

## Supporting Information

S1 Fig The consistency of direct and indirect estimates. When RoR values close to 1, direct and indirect estimates could be considered in agreement. PVP: Percutaneous vertebroplasty; BK: Balloon kyphoplasty; CT: Conservative treatment.
(EPS)

S1 File The key assumption that the three treatment effects originate from one common distribution. (DOC)

S2 File A list of 137 excluded studies and the reasons. (DOC)

S3 File Sensitivity analysis by excluding a study with high risk of bias. (DOC)

S1 PRISMA Checklist PRISMA Checklist for this meta-analysis. (DOC)

S1 Table Sensitivity analysis by excluding a study with different duration of follow-up. For VAS, standard mean differences (SMDs) lower than 0 favour the column-defining treatment. Direct comparsions were shown in the upper right. Indirect comparsions were shown in the bottom left. The number which was painted by a style of overstriking indicated there was a significant difference between two treatments. PVP: Percutaneous vertebroplasty; BK: Balloon kyphoplasty; CT: Conservative treatment. On the basis of SUCRA, percutaneous vertebroplasty (0.8837) ranked the first, the second was balloon kyphoplasty (0.5702) and the last was conservative treatment (0.0461).
(DOC)
