# Supplementary material for: Comparative Efficacy and Tolerability of Three Treatments in Old People with Osteoporotic Vertebral Compression Fracture: A Network Meta-Analysis and Systematic Review
Source: PLoS One. 2015 Apr 13;10(4):e0123153. doi: 10.1371/journal.pone.0123153 (PMC4395314; doi:10.1371/journal.pone.0123153)
Supplement: S1 File — (DOC) [file pone.0123153.s002.doc]

**S1 File. The key assumption that the three treatment effects originate from one common distribution.**

For clinical diversity, there is no obvious difference among five included studies. Firstly, the characteristics of included patients are almost the same: all patients have vertebral fracture whose location is in thoracic and lumbar vertebra; the mean age of the patients are larger than 60; the number of female is larger than male. Secondly, although interventions referred in different studies are not the same, the outcome measurements are the same: VAS score in all studies ranges from 0 (no pain) to 10 (worst pain), the measurement of risk of all-cause discontinuation and incidence of new fractures is the same.

For methodological diversity, two studies have high risk of bias, however, one study is the only included study in three outcomes. So we make a sensitivity analysis by excluding another study. The results are as follows:

VAS:

Before exclude Klazen 2010 -1.81 (-3.1, -0.47)

After exclude Klazen 2010 -1.97 (-4.6, 0.64)

Incidence of new fractures:

Before exclude Klazen 2010 2.05 (0.92, 4.6)

After exclude Klazen 2010 2.4 (0.95, 6.06)

Risk of all-cause discontinuation

Before exclude Klazen 2010 0.9 (0.53, 1.5)

After exclude Klazen 2010 1.35 (0.63, 2.88)

Although the result for VAS has changed to no significant, the CIs are almost overlapped. Other results are almost the same. As a result, no obvious differences are found in all included studies for methodological diversity.

For statistical heterogeneity, the single study’s result and synthetic results for three outcomes are almost overlapped. Only one study’s result for VAS is not overlapped from others, however, the result is the same with others which favor percutaneous vertebroplasty comparing with conservative treatment.

From above three aspects, we think the three treatment effects originate from one common distribution.
