# Supplementary material for: Comparative Efficacy and Tolerability of Three Treatments in Old People with Osteoporotic Vertebral Compression Fracture: A Network Meta-Analysis and Systematic Review
Source: PLoS One. 2015 Apr 13;10(4):e0123153. doi: 10.1371/journal.pone.0123153 (PMC4395314; doi:10.1371/journal.pone.0123153)
Supplement: S2 File — (DOC) [file pone.0123153.s003.doc]

**S2 File. A list of 137 excluded studies and the reasons**

1. Yimin Y, Zhi Z, ZhiWei R, Wei M, Jha RK (2014) Applications of memory alloy stent in vertebral fractures. Med Sci Monit Basic Res 20: 76-81.

**Did not report outcomes that met inclusion criteria**

2. Xu Y, Li B, Zou H, Sheng H, Wang W, et al. (2014) [Imaging assessing symptomatic vertebral compression fracture to be treated by vertebroplasty and kyphoplasty in osteoporosis patients]. Zhonghua Yi Xue Za Zhi 94: 832-835.

**Did not report outcomes that met inclusion criteria**

3. Teyssedou S, Saget M, Pries P (2014) Kyphopasty and vertebroplasty. Orthop Traumatol Surg Res 100: S169-179.

**Review article**

4. Syrimpeis V, Vitsas V, Korovessis P (2014) Lumbar vertebral hemangioma mimicking lateral spinal canal stenosis: case report and review of literature. J Spinal Cord Med 37: 237-242.

**Review article**

5. Pirris SM, Kimes SM (2014) Compression fracture in the middle of a chronic instrumented fusion that developed into pseudarthrosis after balloon kyphoplasty. J Neurosurg Spine 20: 705-708.

6. Civelek E, Cansever T, Yilmaz C, Kabatas S, Gulsen S, et al. (2014) The retrospective analysis of the effect of balloon kyphoplasty to the adjacent-segment fracture in 171 patients. J Spinal Disord Tech 27: 98-104.

**Retrospective design**

7. Bornemann R, Koch EM, Wollny M, Pflugmacher R (2014) Treatment options for vertebral fractures an overview of different philosophies and techniques for vertebral augmentation. Eur J Orthop Surg Traumatol 24 Suppl 1: S131-143.

**Review article**

8. Zhou J, Zhang Z, Huasong M, Tan R, Zou D (2013) Clinical evaluation of Crosstrees pod kyphoplasty in the treatment of osteoporotic vertebral compression fractures. Acta Orthop Belg 79: 451-456.

**Did not report outcomes that met inclusion criteria**

9. Yokoyama K, Kawanishi M, Yamada M, Tanaka H, Ito Y, et al. (2013) In not only vertebroplasty but also kyphoplasty, the resolution of vertebral deformities depends on vertebral mobility. AJNR Am J Neuroradiol 34: 1474-1478.

**Non-randomized design**

10. Yimin Y, Zhiwei R, Wei M, Jha R (2013) Current status of percutaneous vertebroplasty and percutaneous kyphoplasty--a review. Med Sci Monit 19: 826-836.

**Review article**

11. Xing D, Ma JX, Ma XL, Wang J, Xu WG, et al. (2013) A meta-analysis of balloon kyphoplasty compared to percutaneous vertebroplasty for treating osteoporotic vertebral compression fractures. J Clin Neurosci 20: 795-803.

**Meta-analysis**

12. Wang Y, Huang F, Chen L, Ke ZY, Deng ZL (2013) Clinical measurement of intravertebral pressure during vertebroplasty and kyphoplasty. Pain Physician 16: E411-418.

**Did not report outcomes that met inclusion criteria**

13. Wang D, Dai WX, Feng J, Ma C, Wu DH (2013) [Efficacy evaluation of percutaneous vertebroplasty via an extra-pedicular approach for osteoporotic compression fracture]. Zhonghua Yi Xue Za Zhi 93: 845-848.

**Did not report outcomes that met inclusion criteria**

14. Sun G, Wei D, Liu X, Chen Y, Li M, et al. (2013) Novel biodegradable electrospun nanofibrous P(DLLA-CL) balloons for the treatment of vertebral compression fractures. Nanomedicine 9: 829-838.

**Non-randomized design**

15. Rebolledo BJ, Gladnick BP, Unnanuntana A, Nguyen JT, Kepler CK, et al. (2013) Comparison of unipedicular and bipedicular balloon kyphoplasty for the treatment of osteoporotic vertebral compression fractures: a prospective randomised study. Bone Joint J 95-B: 401-406.

**There was no control group that met inclusion criteria**

16. Qian L, Pan J, Liu ZD, Li LJ, Tan J, et al. (2013) The correlation between vertebral wedge-shaped changes in X-ray imaging at supine and standing positions and the efficacy of operative treatment of thoracolumbar spinal fracture in the elderly. Spinal Cord 51: 904-908.

**Did not report outcomes that met inclusion criteria**

17. Ohba T, Ebata S, Clinton D, Koyama K, Haro H (2013) Instability of treated vertebrae after balloon kyphoplasty causing paraparesis in osteoporotic vertebral compression fracture: a report of two cases. Eur Spine J 22 Suppl 3: S341-345.

**Case report**

18. Lin J, Zhang L, Yang HL (2013) Unilateral versus bilateral balloon kyphoplasty for osteoporotic vertebral compression fractures. Pain Physician 16: 447-453.

**There was no control group that met inclusion criteria**

19. Labbe JL, Peres O, Leclair O, Goulon R, Scemama P, et al. (2013) Minimally invasive treatment of displaced intra-articular calcaneal fractures using the balloon kyphoplasty technique: preliminary study. Orthop Traumatol Surg Res 99: 829-836.

**Non-randomized design**

20. Kruger A, Baroud G, Noriega D, Figiel J, Dorschel C, et al. (2013) Height restoration and maintenance after treating unstable osteoporotic vertebral compression fractures by cement augmentation is dependent on the cement volume used. Clin Biomech (Bristol, Avon) 28: 725-730.

**Non-randomized design**

21. Jansen T, Bornemann R, Otten LA, Kabir K, Wirtz D, et al. (2013) [Radiofrequency kyphoplasty combined with posterior fixation in the treatment of burst fractures]. Z Orthop Unfall 151: 632-637.

**Did not report outcomes that met inclusion criteria**

22. Hsieh MK, Chen LH, Chen WJ (2013) Current concepts of percutaneous balloon kyphoplasty for the treatment of osteoporotic vertebral compression fractures: evidence-based review. Biomed J 36: 154-161.

**Review article**

23. Georgy BA (2013) Comparison between radiofrequency targeted vertebral augmentation and balloon kyphoplasty in the treatment of vertebral compression fractures: addressing factors that affect cement extravasation and distribution. Pain Physician 16: E513-518.

**Did not report outcomes that met inclusion criteria**

24. Ge Z, Ma R, Chen Z, Zhang H, Ding H, et al. (2013) Uniextrapedicular kyphoplasty for the treatment of thoracic osteoporotic vertebral fractures. Orthopedics 36: e1020-1024.

**Did not report outcomes that met inclusion criteria**

25. Erdem E, Akdol S, Amole A, Fryar K, Eberle RW (2013) Radiofrequency-targeted vertebral augmentation for the treatment of vertebral compression fractures as a result of multiple myeloma. Spine (Phila Pa 1976) 38: 1275-1281.

**Included non-osteoporotic population**

26. Chitale A, Prasad S (2013) An evidence-based analysis of vertebroplasty and kyphoplasty. J Neurosurg Sci 57: 129-137.

**Meta-analysis**

27. Zarate B, Gutierrez J, Wakhloo AK, Gounis MJ, Reyes-Sanchez A (2012) Clinical evaluation of a new kyphoplasty technique with directed cement flow. J Spinal Disord Tech 25: E61-66.

**Non-randomized design**

28. Zafeiris CP, Lyritis GP, Papaioannou NA, Gratsias PE, Galanos A, et al. (2012) Hypovitaminosis D as a risk factor of subsequent vertebral fractures after kyphoplasty. Spine J 12: 304-312.

**Did not report outcomes that met inclusion criteria**

29. Wu AM, Ni WF, Weng W, Chi YL, Xu HZ, et al. (2012) Outcomes of percutaneous kyphoplasty in patients with intravertebral vacuum cleft. Acta Orthop Belg 78: 790-795.

**Included non-osteoporotic population**

30. Wilson DC, Connolly RJ, Zhu Q, Emery JL, Kingwell SP, et al. (2012) An ex vivo biomechanical comparison of a novel vertebral compression fracture treatment system to kyphoplasty. Clin Biomech (Bristol, Avon) 27: 346-353.

**Non-randomized design**

31. Wardlaw D, Van Meirhaeghe J, Ranstam J, Bastian L, Boonen S (2012) Balloon kyphoplasty in patients with osteoporotic vertebral compression fractures. Expert Rev Med Devices 9: 423-436.

**Review article**

32. Wang Z, Wang G, Yang H (2012) Comparison of unilateral versus bilateral balloon kyphoplasty for the treatment of osteoporotic vertebral compression fractures. J Clin Neurosci 19: 723-726.

**There was no control group that met inclusion criteria**

33. Walter J, Haciyakupoglu E, Waschke A, Kalff R, Ewald C (2012) Cement leakage as a possible complication of balloon kyphoplasty--is there a difference between osteoporotic compression fractures (AO type A1) and incomplete burst fractures (AO type A3.1)? Acta Neurochir (Wien) 154: 313-319.

**Did not report outcomes that met inclusion criteria**

34. Teyssedou S, Saget M, Prebet R, Leclercq N, Vendeuvre T, et al. (2012) Evaluation of percutaneous surgery in the treatment of thoracolumbar fractures. Preliminary results of a prospective study on 65 patients. Orthop Traumatol Surg Res 98: 39-47.

**Non-randomized design**

35. Sandmann GH, Ahrens P, Schaeffeler C, Bauer JS, Kirchhoff C, et al. (2012) Balloon osteoplasty--a new technique for minimally invasive reduction and stabilisation of Hill-Sachs lesions of the humeral head: a cadaver study. Int Orthop 36: 2287-2291.

**Non-randomized design**

36. Qian J, Yang H, Jing J, Zhao H, Ni L, et al. (2012) The early stage adjacent disc degeneration after percutaneous vertebroplasty and kyphoplasty in the treatment of osteoporotic VCFs. PLoS One 7: e46323.

**Did not report outcomes that met inclusion criteria**

37. Pflugmacher R, Bornemann R, Koch EM, Randau TM, Muller-Broich J, et al. (2012) [Comparison of clinical and radiological data in the treatment of patients with osteoporotic vertebral compression fractures using radiofrequency kyphoplasty or balloon kyphoplasty]. Z Orthop Unfall 150: 56-61.

**Did not report outcomes that met inclusion criteria**

38. Papanastassiou ID, Aghayev K, Berenson JR, Schmidt MH, Vrionis FD (2012) Is vertebral augmentation the right choice for cancer patients with painful vertebral compression fractures? J Natl Compr Canc Netw 10: 715-719.

**Included non-osteoporotic population**

39. Ondul S, Durmus M (2012) Minimally invasive stabilization of vertebral compression fractures using balloon kyphoplasty. J Neurosurg Sci 56: 357-361.

**Did not report outcomes that met inclusion criteria**

40. Movrin I (2012) Adjacent level fracture after osteoporotic vertebral compression fracture: a nonrandomized prospective study comparing balloon kyphoplasty with conservative therapy. Wien Klin Wochenschr 124: 304-311.

**Non-randomized design**

41. Ma XL, Xing D, Ma JX, Xu WG, Wang J, et al. (2012) Balloon kyphoplasty versus percutaneous vertebroplasty in treating osteoporotic vertebral compression fracture: grading the evidence through a systematic review and meta-analysis. Eur Spine J 21: 1844-1859.

**Meta-analysis**

42. Lee MJ, Jarvik JG (2012) [Cement augmentation for osteoporotic compression fractures]. Unfallchirurg 115: 1066-1069.

**Review article**

43. Katonis P, Hadjipavlou A, Souvatzis X, Tzermiadianos M, Alpantaki K, et al. (2012) Respiratory effects, hemodynamic changes and cement leakage during multilevel cement balloon kyphoplasty. Eur Spine J 21: 1860-1866.

**Did not report outcomes that met inclusion criteria**

44. Itshayek E, Miller P, Barzilay Y, Hasharoni A, Kaplan L, et al. (2012) Vertebral augmentation in the treatment of vertebral compression fractures: review and new insights from recent studies. J Clin Neurosci 19: 786-791.

**Review article**

45. Edidin AA, Ong KL, Lau E, Schmier JK, Kemner JE, et al. (2012) Cost-effectiveness analysis of treatments for vertebral compression fractures. Appl Health Econ Health Policy 10: 273-284.

**Cost-effectiveness analysis**

46. Dalton BE, Kohm AC, Miller LE, Block JE, Poser RD (2012) Radiofrequency-targeted vertebral augmentation versus traditional balloon kyphoplasty: radiographic and morphologic outcomes of an ex vivo biomechanical pilot study. Clin Interv Aging 7: 525-531.

**Non-randomized design**

47. Bergmann M, Oberkircher L, Bliemel C, Frangen TM, Ruchholtz S, et al. (2012) Early clinical outcome and complications related to balloon kyphoplasty. Orthop Rev (Pavia) 4: e25.

**Did not report outcomes that met inclusion criteria**

48. Robinson Y, Heyde CE, Forsth P, Olerud C (2011) Kyphoplasty in osteoporotic vertebral compression fractures--guidelines and technical considerations. J Orthop Surg Res 6: 43.

**Review article**

49. Robertson SC (2011) Percutaneous vertebral augmentation: StabilitiT a new delivery system for vertebral fractures. Acta Neurochir Suppl 108: 191-195.

**Non-randomized design**

50. Prokop A, Konig B, Schultheiss M, Andresen R (2011) [Kyphoplasty update : What are the limits - what is possible?]. Unfallchirurg 114: 1035-1040.

**Review article**

51. Piazzolla A, De Giorgi G, Solarino G (2011) Vertebral body recollapse without trauma after kyphoplasty with calcium phosphate cement. Musculoskelet Surg 95: 141-145.

**Did not report outcomes that met inclusion criteria**

52. Mohr M, Pillich D, Kirsch M, Mueller JU, Fleck S, et al. (2011) Percutaneous balloon kyphoplasty with the patient under intravenous analgesia and sedation: a feasibility study. AJNR Am J Neuroradiol 32: 649-653.

**Included non-osteoporotic population**

53. Lim BG, Lee JY, Lee MK, Lee DK, Kim JS, et al. (2011) Kyphoplasty for the treatment of vertebral compression fractures in a cancer patient with neurological deficits and anterior vertebral wall destruction. Pain Physician 14: 539-544.

**Included non-osteoporotic population**

54. La Maida GA, Sala F, Callea G, Capitani D, Singh S (2011) Efficacy of unipedicular baloon kyphoplasty for treatment of multiple myeloma vertebral lesions. Asian Spine J 5: 162-168.

**Included non-osteoporotic population**

55. Han S, Wan S, Ning L, Tong Y, Zhang J, et al. (2011) Percutaneous vertebroplasty versus balloon kyphoplasty for treatment of osteoporotic vertebral compression fracture: a meta-analysis of randomised and non-randomised controlled trials. Int Orthop 35: 1349-1358.

**Meta-analysis**

56. Fritzell P, Ohlin A, Borgstrom F (2011) Cost-effectiveness of balloon kyphoplasty versus standard medical treatment in patients with osteoporotic vertebral compression fracture: a Swedish multicenter randomized controlled trial with 2-year follow-up. Spine (Phila Pa 1976) 36: 2243-2251.

**Cost-effectiveness analysis**

57. Friedrich HC, Friedrich HJ, Kneisel P, Drumm J, Pitzen T (2011) Balloon kyphoplasty improves back pain but does not result in a permanent realignment of the thoracolumbar spine. Cent Eur Neurosurg 72: 176-180.

**Non-randomized design**

58. Eleraky M, Papanastassiou I, Setzer M, Baaj AA, Tran ND, et al. (2011) Balloon kyphoplasty in the treatment of metastatic tumors of the upper thoracic spine. J Neurosurg Spine 14: 372-376.

**Included non-osteoporotic population**

59. Chen L, Yang H, Tang T (2011) Unilateral versus bilateral balloon kyphoplasty for multilevel osteoporotic vertebral compression fractures: a prospective study. Spine (Phila Pa 1976) 36: 534-540.

**There was no control group that met inclusion criteria**

60. Chatziioannou SN, Savvidou C, Pianou NK, Athanassacopoulos M, Pneumaticos SG (2011) Balloon kyphoplasty: scintigraphy as the ultimate decision maker? Acta Orthop Belg 77: 371-374.

**Review article**

61. Aghayev K, Papanastassiou ID, Vrionis F (2011) Role of vertebral augmentation procedures in the management of vertebral compression fractures in cancer patients. Curr Opin Support Palliat Care 5: 222-226.

**Included non-osteoporotic population**

62. Zhao YL, Yang HL, Konrad J, Liu J, Moral M, et al. (2010) Kyphoplasty does not maintain all restored height postoperatively: a prospective, comparative study. Orthopedics 33.

**Non-randomized design**

63. Zampini JM, White AP, McGuire KJ (2010) Comparison of 5766 vertebral compression fractures treated with or without kyphoplasty. Clin Orthop Relat Res 468: 1773-1780.

**Retrospective design**

64. Wick M, Petraschka C, Kronawitter P, Cidlinsky K, Heyer C (2010) [Osteoporotic vertebral fractures in the elderly: are conventional radiographs useful? - clinical and radiographic results after kyphoplasty]. Z Orthop Unfall 148: 641-645.

**Did not report outcomes that met inclusion criteria**

65. Wang GL, Yang HL, Jiang WM, Chen L, Meng B, et al. (2010) [Balloon kyphoplasty for osteoporotic vertebral compression fractures with osteonecrosis]. Zhonghua Wai Ke Za Zhi 48: 593-596.

**Included non-osteoporotic population**

66. Wang G, Yang H, Chen K (2010) Osteoporotic vertebral compression fractures with an intravertebral cleft treated by percutaneous balloon kyphoplasty. J Bone Joint Surg Br 92: 1553-1557.

**Non-randomized design**

67. Upasani VV, Robertson C, Lee D, Tomlinson T, Mahar AT (2010) Biomechanical comparison of kyphoplasty versus a titanium mesh implant with cement for stabilization of vertebral compression fractures. Spine (Phila Pa 1976) 35: 1783-1788.

**Non-randomized design**

68. Strom O, Leonard C, Marsh D, Cooper C (2010) Cost-effectiveness of balloon kyphoplasty in patients with symptomatic vertebral compression fractures in a UK setting. Osteoporos Int 21: 1599-1608.

**Cost-effectiveness analysis**

69. Ryu KS, Shim JH, Heo HY, Park CK (2010) Therapeutic efficacy of injectable calcium phosphate cement in osteoporotic vertebral compression fractures: prospective nonrandomized controlled study at 6-month follow-up. World Neurosurg 73: 408-411.

**Non-randomized design**

70. Rotter R, Martin H, Fuerderer S, Gabl M, Roeder C, et al. (2010) Vertebral body stenting: a new method for vertebral augmentation versus kyphoplasty. Eur Spine J 19: 916-923.

**Non-randomized design**

71. Park SY, Modi HN, Suh SW, Hong JY, Noh W, et al. (2010) Epidural cement leakage through pedicle violation after balloon kyphoplasty causing paraparesis in osteoporotic vertebral compression fractures - a report of two cases. J Orthop Surg Res 5: 54.

**Case report**

72. Ortiz O, Mathis JM (2010) Vertebral body reconstruction: techniques and tools. Neuroimaging Clin N Am 20: 145-158.

**Non-randomized design**

73. Muller CW, Gosling T, Mameghani A, Stier R, Klein M, et al. (2010) [Vertebral fractures due to osteoporosis. Kyphoplasty and vertebroplasty vs conservative treatment]. Orthopade 39: 417-424.

**Non-randomized design**

74. Movrin I, Vengust R, Komadina R (2010) Adjacent vertebral fractures after percutaneous vertebral augmentation of osteoporotic vertebral compression fracture: a comparison of balloon kyphoplasty and vertebroplasty. Arch Orthop Trauma Surg 130: 1157-1166.

**Did not report outcomes that met inclusion criteria**

75. Limthongkul W, Karaikovic EE, Savage JW, Markovic A (2010) Volumetric analysis of thoracic and lumbar vertebral bodies. Spine J 10: 153-158.

**Non-randomized design**

76. Li Y, Lewis G (2010) Influence of loading cycle profile and frequency on a biomechanical parameter of a model of a balloon kyphoplasty-augmented lumbar spine segment: a finite element analysis study. Biomed Mater Eng 20: 349-359.

**Non-randomized design**

77. Lewis G, Towler MR, Boyd D, German MJ, Wren AW, et al. (2010) Evaluation of two novel aluminum-free, zinc-based glass polyalkenoate cements as alternatives to PMMA bone cement for use in vertebroplasty and balloon kyphoplasty. J Mater Sci Mater Med 21: 59-66.

**Non-randomized design**

78. Kruger A, Zettl R, Ziring E, Mann D, Schnabel M, et al. (2010) Kyphoplasty for the treatment of incomplete osteoporotic burst fractures. Eur Spine J 19: 893-900.

**Included non-osteoporotic population**

79. Kim YY, Rhyu KW (2010) Recompression of vertebral body after balloon kyphoplasty for osteoporotic vertebral compression fracture. Eur Spine J 19: 1907-1912.

**Did not report outcomes that met inclusion criteria**

80. Kim HS, Kim SH, Ju CI, Kim SW, Lee SM, et al. (2010) The role of bone cement augmentation in the treatment of chronic symptomatic osteoporotic compression fracture. J Korean Neurosurg Soc 48: 490-495.

**Did not report outcomes that met inclusion criteria**

81. Kasper DM (2010) Kyphoplasty. Semin Intervent Radiol 27: 172-184.

**Review article**

82. Hillmeier J (2010) [Balloon kyphoplasty]. Orthopade 39: 665-672.

**Review article**

83. Health Quality O (2010) Percutaneous vertebroplasty for treatment of painful osteoporotic vertebral compression fractures: an evidence-based analysis. Ont Health Technol Assess Ser 10: 1-45.

**Meta-analysis**

84. Hadley C, Awan OA, Zoarski GH (2010) Biomechanics of vertebral bone augmentation. Neuroimaging Clin N Am 20: 159-167.

**Non-randomized design**

85. Guo J, Ding W, Shen Y, Li B, Wu H, et al. (2010) [Selective treatment of aged osteoporosis thoracolumbar vertebrae burst fracture with balloon kyphoplasty]. Zhongguo Xiu Fu Chong Jian Wai Ke Za Zhi 24: 1341-1344.

**Non-randomized design**

86. Ghofrani H, Nunn T, Robertson C, Mahar A, Lee Y, et al. (2010) An evaluation of fracture stabilization comparing kyphoplasty and titanium mesh repair techniques for vertebral compression fractures: is bone cement necessary? Spine (Phila Pa 1976) 35: E768-773.

**Did not report outcomes that met inclusion criteria**

87. Dong Y, Wang DY (2010) [Treatment of osteoporotic vertebral compression fractures by ballon kyphoplasty]. Zhongguo Gu Shang 23: 466-467.

**Non-randomized design**

88. Dalbayrak S, Onen MR, Yilmaz M, Naderi S (2010) Clinical and radiographic results of balloon kyphoplasty for treatment of vertebral body metastases and multiple myelomas. J Clin Neurosci 17: 219-224.

**Included non-osteoporotic population**

89. Bula P, Lein T, Strassberger C, Bonnaire F (2010) [Balloon kyphoplasty in the treatment of osteoporotic vertebral fractures: indications - treatment strategy - complications]. Z Orthop Unfall 148: 646-656.

**Did not report outcomes that met inclusion criteria**

90. Wang J, Zhang N, Liu J, Tian H, Chen C (2009) [Clinical study on one side approach percutaneous kyphoplasty treatment of severe osteoporotic vertebral compression fractures]. Zhongguo Xiu Fu Chong Jian Wai Ke Za Zhi 23: 68-71.

**There was no control group that met inclusion criteria**

91. Song BK, Eun JP, Oh YM (2009) Clinical and radiological comparison of unipedicular versus bipedicular balloon kyphoplasty for the treatment of vertebral compression fractures. Osteoporos Int 20: 1717-1723.

**There was no control group that met inclusion criteria**

92. Pflugmacher R, Agarwal A, Kandziora F, C KK (2009) Balloon kyphoplasty combined with posterior instrumentation for the treatment of burst fractures of the spine--1-year results. J Orthop Trauma 23: 126-131.

**There was no control group that met inclusion criteria**

93. Lewis G, Koole LH, van Hooy-Corstjens CS (2009) Influence of powder-to-liquid monomer ratio on properties of an injectable iodine-containing acrylic bone cement for vertebroplasty and balloon kyphoplasty. J Biomed Mater Res B Appl Biomater 91: 537-544.

**Did not report outcomes that met inclusion criteria**

94. Lee MJ, Dumonski M, Cahill P, Stanley T, Park D, et al. (2009) Percutaneous treatment of vertebral compression fractures: a meta-analysis of complications. Spine (Phila Pa 1976) 34: 1228-1232.

**Meta-analysis**

95. Langdon J, Bernard J, Molloy S (2009) Prophylactic stabilization of vertebral body metastasis at risk of imminent fracture using balloon kyphoplasty. Spine (Phila Pa 1976) 34: E469-472.

**Included non-osteoporotic population**

96. Hamady M, Sheard S (2009) Role of cementoplasty in the management of compression vertebral body fractures. Postgrad Med J 85: 293-298.

**There was no control group that met inclusion criteria**

97. Gerszten PC, Monaco EA, 3rd (2009) Complete percutaneous treatment of vertebral body tumors causing spinal canal compromise using a transpedicular cavitation, cement augmentation, and radiosurgical technique. Neurosurg Focus 27: E9.

**Did not report outcomes that met inclusion criteria**

98. Chen L, Yang HL, Tang TS (2009) [Unilateral versus bilateral balloon kyphoplasty in the treatment of multi-vertebral osteoporotic compression fractures]. Zhonghua Wai Ke Za Zhi 47: 1642-1646.

**Did not report outcomes that met inclusion criteria**

99. Carbognin G, Sandri A, Girardi V, Regis D, Calciolari C, et al. (2009) Treatment of type-A3 amyelic thoracolumbar fractures (burst fractures) with kyphoplasty: initial experience. Radiol Med 114: 133-140.

**Non-randomized design**

100. Blondel B, Fuentes S, Metellus P, Adetchessi T, Pech-Gourg G, et al. (2009) Severe thoracolumbar osteoporotic burst fractures: treatment combining open kyphoplasty and short-segment fixation. Orthop Traumatol Surg Res 95: 359-364.

**There was no control group that met inclusion criteria**

101. Blattert TR, Jestaedt L, Weckbach A (2009) Suitability of a calcium phosphate cement in osteoporotic vertebral body fracture augmentation: a controlled, randomized, clinical trial of balloon kyphoplasty comparing calcium phosphate versus polymethylmethacrylate. Spine (Phila Pa 1976) 34: 108-114.

**There was no control group that met inclusion criteria**

102. Ayvaz M, Alanay A, Acaroglu RE (2009) Minimal invasive short posterior instrumentation plus balloon kyphoplasty with calcium phosphate for burst and severe compression lumbar fractures. Spine (Phila Pa 1976) 34: 2473; author reply 2473-2475.

**There was no control group that met inclusion criteria**

103. Sun G, Jin P, Li FD, Liu XW, Hao RS, et al. (2008) Preliminary study on a single balloon cross-midline expansion via unipedicular approach in kyphoplasty. Chin Med J (Engl) 121: 1811-1814.

**Non-randomized design**

104. Pflugmacher R, Taylor R, Agarwal A, Melcher I, Disch A, et al. (2008) Balloon kyphoplasty in the treatment of metastatic disease of the spine: a 2-year prospective evaluation. Eur Spine J 17: 1042-1048.

**Included non-osteoporotic population**

105. Peh WC, Munk PL, Rashid F, Gilula LA (2008) Percutaneous vertebral augmentation: vertebroplasty, kyphoplasty and skyphoplasty. Radiol Clin North Am 46: 611-635, vii.

**Did not report outcomes that met inclusion criteria**

106. McCall T, Cole C, Dailey A (2008) Vertebroplasty and kyphoplasty: a comparative review of efficacy and adverse events. Curr Rev Musculoskelet Med 1: 17-23.

**Review article**

107. Kwon YJ (2008) Modified vertebroplasty using a curved probe: technique and preliminary results. Minim Invasive Neurosurg 51: 187-191.

**Non-randomized design**

108. Korovessis P, Hadjipavlou A, Repantis T (2008) Minimal invasive short posterior instrumentation plus balloon kyphoplasty with calcium phosphate for burst and severe compression lumbar fractures. Spine (Phila Pa 1976) 33: 658-667.

**Included non-osteoporotic population**

109. Khanna AJ, Lee S, Villarraga M, Gimbel J, Steffey D, et al. (2008) Biomechanical evaluation of kyphoplasty with calcium phosphate cement in a 2-functional spinal unit vertebral compression fracture model. Spine J 8: 770-777.

**Non-randomized design**

110. Taylor RS, Fritzell P, Taylor RJ (2007) Balloon kyphoplasty in the management of vertebral compression fractures: an updated systematic review and meta-analysis. Eur Spine J 16: 1085-1100.

**Meta-analysis**

111. Runge M, Bonneville JF (2007) [Balloon assisted kyphoplasty: new technique for treatment of vertebral compression fractures]. J Radiol 88: 1200-1202.

**Non-randomized design**

112. Olivier E, Beldame J, Ould-Slimane M, Puech N, Lefebvre B, et al. (2007) [Treatment of thoracolumbar junction burst fractures (Magerl A3) by balloon kyphoplasty: anatomic study]. Rev Chir Orthop Reparatrice Appar Mot 93: 666-673.

**Non-randomized design**

113. Frankel BM, Monroe T, Wang C (2007) Percutaneous vertebral augmentation: an elevation in adjacent-level fracture risk in kyphoplasty as compared with vertebroplasty. Spine J 7: 575-582.

**Did not report outcomes that met inclusion criteria**

114. Foo LS, Yeo W, Fook S, Guo CM, Chen JL, et al. (2007) Results, experience and technical points learnt with use of the SKy Bone Expander kyphoplasty system for osteoporotic vertebral compression fractures: a prospective study of 40 patients with a minimum of 12 months of follow-up. Eur Spine J 16: 1944-1950.

**Non-randomized design**

115. De Negri P, Tirri T, Paternoster G, Modano P (2007) Treatment of painful osteoporotic or traumatic vertebral compression fractures by percutaneous vertebral augmentation procedures: a nonrandomized comparison between vertebroplasty and kyphoplasty. Clin J Pain 23: 425-430.

**Non-randomized design**

116. Zhang DJ, Chen JT, Jin DD (2006) [Kyphoplasty using an enhanced balloon expander: an experimental study]. Nan Fang Yi Ke Da Xue Xue Bao 26: 705-706, 710.

**Non-randomized design**

117. Tong SC, Eskey CJ, Pomerantz SR, Hirsch JA (2006) "SKyphoplasty": a single institution's initial experience. J Vasc Interv Radiol 17: 1025-1030.

**Non-randomized design**

118. Taylor RS, Taylor RJ, Fritzell P (2006) Balloon kyphoplasty and vertebroplasty for vertebral compression fractures: a comparative systematic review of efficacy and safety. Spine (Phila Pa 1976) 31: 2747-2755.

**Review article**

119. Schulte BU, Brucher D, Trompeter M, Remy C, Reimer P (2006) [Balloon-assisted percutaneous vertebroplasty in patients with osteoporotic vertebral body compression fractures--first results]. Rofo 178: 207-213.

**There was no control group that met inclusion criteria**

120. Bouza C, Lopez T, Magro A, Navalpotro L, Amate JM (2006) Efficacy and safety of balloon kyphoplasty in the treatment of vertebral compression fractures: a systematic review. Eur Spine J 15: 1050-1067.

**Review article**

121. Becker S, Meissner J, Bartl R, Bretschneider W, Ogon M (2006) Preliminary results with modified techniques of balloon kyphoplasty for vertebra plana, traumatic fractures and neoplasms. Acta Orthop Belg 72: 187-193.

**Non-randomized design**

122. Hu MM, Eskey CJ, Tong SC, Nogueira RG, Pomerantz SR, et al. (2005) Kyphoplasty for vertebral compression fracture via a uni-pedicular approach. Pain Physician 8: 363-367.

**There was no control group that met inclusion criteria**

123. Hadjipavlou AG, Tzermiadianos MN, Katonis PG, Szpalski M (2005) Percutaneous vertebroplasty and balloon kyphoplasty for the treatment of osteoporotic vertebral compression fractures and osteolytic tumours. J Bone Joint Surg Br 87: 1595-1604.

**Included non-osteoporotic population**

124. Truumees E, Hilibrand A, Vaccaro AR (2004) Percutaneous vertebral augmentation. Spine J 4: 218-229.

**Review article**

125. Hoh BL, Rabinov JD, Pryor JC, Hirsch JA (2004) Balloon kyphoplasty for vertebral compression fracture using a unilateral balloon tamp via a uni-pedicular approach: technical note. Pain Physician 7: 111-114.

**Non-randomized design**

126. Health Quality O (2004) Balloon kyphoplasty: an evidence-based analysis. Ont Health Technol Assess Ser 4: 1-45.

**Meta-analysis**

127. Grohs JG, Krepler P (2004) [Minimal invasive stabilization of osteoporotic vertebral compression fractures. Methods and preinterventional diagnostics]. Radiologe 44: 254-259.

**Did not report outcomes that met inclusion criteria**

128. Boszczyk BM, Bierschneider M, Hauck S, Vastmans J, Potulski M, et al. (2004) [Conventional and semi-open kyphoplasty]. Orthopade 33: 13-21.

**Non-randomized design**

129. Wilhelm K, Stoffel M, Ringel F, Rao G, Rosseler L, et al. (2003) [Preliminary experience with balloon kyphoplasty for the treatment of painful osteoporotic compression fractures]. Rofo 175: 1690-1696.

**Non-randomized design**

130. Franck H, Boszczyk BM, Bierschneider M, Jaksche H (2003) Interdisciplinary approach to balloon kyphoplasty in the treatment of osteoporotic vertebral compression fractures. Eur Spine J 12 Suppl 2: S163-167.

**Did not report outcomes that met inclusion criteria**

131. Theodorou DJ, Theodorou SJ, Duncan TD, Garfin SR, Wong WH (2002) Percutaneous balloon kyphoplasty for the correction of spinal deformity in painful vertebral body compression fractures. Clin Imaging 26: 1-5.

**Did not report outcomes that met inclusion criteria**

132. Phillips FM, Todd Wetzel F, Lieberman I, Campbell-Hupp M (2002) An in vivo comparison of the potential for extravertebral cement leak after vertebroplasty and kyphoplasty. Spine (Phila Pa 1976) 27: 2173-2178; discussion 2178-2179.

**Non-randomized design**

133. Ortiz AO, Zoarski GH, Beckerman M (2002) Kyphoplasty. Tech Vasc Interv Radiol 5: 239-249.

**Non-randomized design**

134. Hardouin P, Fayada P, Leclet H, Chopin D (2002) Kyphoplasty. Joint Bone Spine 69: 256-261.

**Non-randomized design**

135. Garfin SR, Reilley MA (2002) Minimally invasive treatment of osteoporotic vertebral body compression fractures. Spine J 2: 76-80.

**Non-randomized design**

136. Boszczyk B, Bierschneider M, Potulski M, Robert B, Vastmans J, et al. (2002) [Extended kyphoplasty indications for stabilization of osteoporotic vertebral compression fractures]. Unfallchirurg 105: 952-957.

**Non-randomized design**

137. Belkoff SM, Mathis JM, Fenton DC, Scribner RM, Reiley ME, et al. (2001) An ex vivo biomechanical evaluation of an inflatable bone tamp used in the treatment of compression fracture. Spine (Phila Pa 1976) 26: 151-156.

**Non-randomized design**

**Non-randomized design (n=45)**

**Did not report outcomes that met inclusion criteria (n=31)**

**Meta-analysis, cost-effectiveness analysis and review article (n=28)**

**Included non-osteoporotic population (n=15)**

**There was no control group that met inclusion criteria (n=13)**

**Retrospective design and case report (n=5)**
