# Supplementary material for: Comparative Efficacy and Tolerability of Three Treatments in Old People with Osteoporotic Vertebral Compression Fracture: A Network Meta-Analysis and Systematic Review
Source: PLoS One. 2015 Apr 13;10(4):e0123153. doi: 10.1371/journal.pone.0123153 (PMC4395314; doi:10.1371/journal.pone.0123153)
Supplement: S3 File — (DOC) [file pone.0123153.s004.doc]

**S3 File. Sensitivity analysis by excluding a study with high risk of bias**

VAS:

Traditional meta-analysis

Before exclude Klazen 2010 -1.81 (-3.1, -0.47)

After exclude Klazen 2010 -1.97 (-4.6, 0.64)

Network meta-analysis

Before exclude Klazen 2010 -1.71 (-2.4, -0.95)

After exclude Klazen 2010 -1.81 (-2.69, -0.89)

Incidence of new fractures:

Traditional meta-analysis

Before exclude Klazen 2010 2.05 (0.92, 4.6)

After exclude Klazen 2010 2.4 (0.95, 6.06)

Network meta-analysis

Before exclude Klazen 2010 2.4 (0.73, 5)

After exclude Klazen 2010 3.2 (0.77, 7.9)

Risk of all-cause discontinuation

Traditional meta-analysis

Before exclude Klazen 2010 0.9 (0.53, 1.5)

After exclude Klazen 2010 1.35 (0.63, 2.88)

Network meta-analysis

Before exclude Klazen 2010 1.04 (0.5, 1.9)

After exclude Klazen 2010 1.68 (0.55, 3.6)

Although the result of traditional meta-analysis for VAS has changed to no significant, the CIs are almost overlapped. Other results are almost the same.
