# Supplementary material for: Comparative Efficacy and Tolerability of Three Treatments in Old People with Osteoporotic Vertebral Compression Fracture: A Network Meta-Analysis and Systematic Review
Source: PLoS One. 2015 Apr 13;10(4):e0123153. doi: 10.1371/journal.pone.0123153 (PMC4395314; doi:10.1371/journal.pone.0123153)
Supplement: S1 Table — For VAS, standard mean differences (SMDs) lower than 0 favour the column-defining treatment. Direct comparsions were shown in the upper right. Indirect comparsions were shown in the bottom left. The number which was painted by a style of overstriking indicated there was a significant difference between two treatments. PVP: Percutaneous vertebroplasty; BK: Balloon kyphoplasty; CT: Conservative treatment. On the basis of SUCRA, percutaneous vertebroplasty (0.8837) ranked the first, the second was balloon kyphoplasty (0.5702) and the last was conservative treatment (0.0461). (DOC) [file pone.0123153.s006.doc]

**S1 Table. Sensitivity analysis by excluding a study with different duration of follow-up.**

| PVP | / | **-1.81(-3.1--0.47)** |
| --- | --- | --- |
| -0.7008(-0.23-0.94) | BK | **-1.1(-1.4--0.89)** |
| **-1.83(-2.63--1)** | -1.13(-2.52-0.28) | CT |

For VAS, standard mean differences (SMDs) lower than 0 favour the column-defining treatment. Direct comparsions were shown in the upper right. Indirect comparsions were shown in the bottom left. The number which was painted by a style of overstriking indicated there was a significant difference between two treatments. PVP：Percutaneous vertebroplasty; BK：Balloon kyphoplasty; CT：Conservative treatment. On the basis of SUCRA, percutaneous vertebroplasty (0.8837) ranked the first, the second was balloon kyphoplasty (0.5702) and the last was conservative treatment (0.0461).
